# Health Gain, Cost Impacts, and Cost-Effectiveness of a Mass Media Campaign to Promote Smartphone Apps for Physical Activity: Modeling Study

**DOI:** 10.2196/18014

**Published:** 2020-06-11

**Authors:** Anja Mizdrak, Kendra Telfer, Artur Direito, Linda J Cobiac, Tony Blakely, Christine L Cleghorn, Nick Wilson

**Affiliations:** 1 Department of Public Health University of Otago Wellington Wellington New Zealand; 2 Yong Loo Lin School of Medicine National University of Singapore Singapore Singapore; 3 Nuffield Department of Population Health University of Oxford Oxford United Kingdom; 4 Centre for Epidemiology and Biostatistics University of Melbourne Melbourne Australia

**Keywords:** physical activity, mHealth, mobile health, smartphone apps, modeling, mass media campaigns

## Abstract

**Background:**

Physical activity smartphone apps are a promising strategy to increase population physical activity, but it is unclear whether government mass media campaigns to promote these apps would be a cost-effective use of public funds.

**Objective:**

We aimed to estimate the health impacts, costs, and cost-effectiveness of a one-off national mass media campaign to promote the use of physical activity apps.

**Methods:**

We used an established multistate life table model to estimate the lifetime health gains (in quality-adjusted life years [QALYs]) that would accrue if New Zealand adults were exposed to a one-off national mass media campaign to promote physical activity app use, with a 1-year impact on physical activity, compared to business-as-usual. A health-system perspective was used to assess cost-effectiveness. and a 3% discount rate was applied to future health gains and health system costs.

**Results:**

The modeled intervention resulted in 28 QALYs (95% uncertainty interval [UI] 8-72) gained at a cost of NZ $81,000/QALY (2018 US $59,500; 95% UI 17,000-345,000), over the remaining life course of the 2011 New Zealand population. The intervention had a low probability (20%) of being cost-effective at a cost-effectiveness threshold of NZ $45,000 (US $32,900) per QALY. The health impact and cost-effectiveness of the intervention were highly sensitive to assumptions around the maintenance of physical activity behaviors beyond the duration of the intervention.

**Conclusions:**

A mass media campaign to promote smartphone apps for physical activity is unlikely to generate much health gain or be cost-effective at the population level. Other investments to promote physical activity, particularly those that result in sustained behavior change, are likely to have greater health impacts.

## Introduction

Insufficient physical activity is associated with an increased risk of cardiovascular diseases, cancers, and poor mental health [1-3]. International recommendations state that adults should aim to accumulate at least 150 minutes of moderate-to-vigorous physical activity (MVPA) throughout the week [1,4]. Prevalence of insufficient physical activity is high in many countries: 40% in the United States, 34% in India, 47% in Brazil, and 42% in New Zealand [5]. Strategies to increase physical activity at the population level are needed, and the promotion of smartphone apps for physical activity is one promising avenue for intervention.

The rise of physical activity smartphone apps provides an opportunity to deliver interventions that have wide reach and a range of technology-enhanced features (eg, accelerometers, tailored feedback, and reminders) [6]. Evaluations of the effectiveness of physical activity apps have shown they can be effective at increasing physical activity levels [7-9]. However, there is high variability in the quality and effectiveness of the thousands of physical activity apps that are currently available [6,10]. Encouraging the use of high-quality apps provides a potential opportunity to increase population-level physical activity owing to the large potential reach and low cost of apps. Additionally, there is growing evidence of the cost-effectiveness of mobile health interventions as a whole [11].

Recent attempts have been made to improve public awareness around the quality and effectiveness of different health apps. Several government agencies around the world now provide app ratings or recommendations on their websites [12-16], but the levels of public engagement have not been publicly documented. Mass media campaigns provide a potential avenue to promote the use of high-quality physical activity apps and, thereby, result in increases in physical activity levels. A recent review suggests that mass media campaigns can be effective, but evidence on the cost-effectiveness is largely limited to tobacco control [17]. Our previous research has assessed the potential of mass media campaigns that promote smartphone apps: a mass media campaign promoting smoking cessation apps is likely to be cost-saving [18], while a mass media campaign for weight loss apps may or may not be cost-effective owing to wide uncertainty around intervention impacts [19]. Although the short-term effectiveness of physical activity apps has been assessed [7,8], it is unknown whether promoting physical activity apps through mass media would be effective or cost-effective. Similarly, we do not know how impacts of mass media campaigns to promote physical activity apps may compare to other public health interventions.

To fill this gap, this study assessed the health impacts, costs, and cost-effectiveness of a mass media campaign to promote high quality smartphone apps for physical activity in a high-income country setting (New Zealand) using a multistate life table modeling approach parameterized with age, sex, and ethnicity specific data consistent with previous work [18,19].

## Methods

### Overview

We used an established proportional multistate life table model to estimate the health impact of a mass media campaign to promote the use of physical activity smartphone apps [20,21]. The model simulates the entire New Zealand population, alive in 2011, out until death under both business-as-usual (BAU) and the modeled intervention. Health gain was measured in quality-adjusted life years (QALYs)—a summary measure of population health that captures both morbidity and mortality impacts to be considered simultaneously [22]. For costs, we used a health-system perspective, and the outputs were the difference in total health system costs (the net sum of intervention costs and downstream cost offsets due to altered disease rates) between BAU and the modeled intervention.

A 3% discount rate was applied to both health gains and health system costs in accordance with prior New Zealand research. Results for 0% and 6% discount rates are presented as scenario analyses. Full details of the model are published elsewhere [20,21].

### Intervention Specification

**Figure 1:**
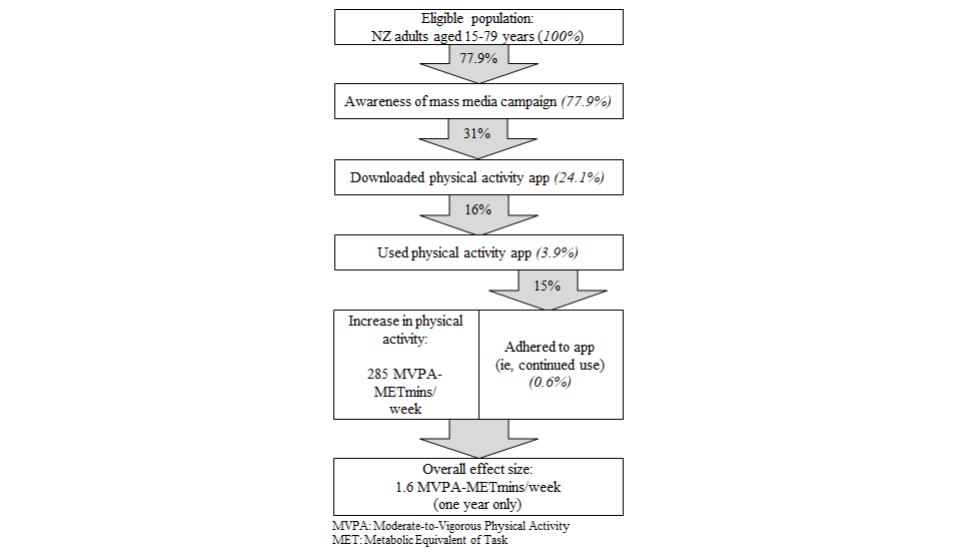
Flow chart of intervention conceptualization. Italicized text represents the percentage of the eligible population exposed to each step in the intervention pathway. NZ: New Zealand.

We modeled a one-off mass media campaign according to the intervention pathway displayed in Figure 1. The population eligible for the intervention included all New Zealand adults 15-79 years of age—the population for which physical activity data were available. Of the population eligible for the intervention, we estimated the proportion of the population that would experience increased physical activity based on likely awareness of the mass media campaign, app download rates, and app use. We defined app use as use for at least 7 days following the initial download of the app to ensure consistency between the modeled intervention pathway and available evidence. Increases in physical activity associated with app use were estimated from a recent systematic review with a meta-analysis of the effectiveness of physical activity apps [8]. Increases in app use wane over time [23], with no evidence of maintained effect beyond 1 year [8]. As our model projects health gains in 1-year time steps, we estimated an average adherence to physical activity apps across the year in which the intervention was implemented [24]. Sources of parameter values are detailed in Table 1, and further detail on parameter selection is available in a related technical report [24].

**Table 1:** Intervention parameters and uncertainty distributions.

Parameter	Value	Distribution	Source
Adult NZ^a^ population aware of mass media campaign, % (UI^b^)	77.9 (70-83)	Beta	Based on awareness of previous health-related mass media campaign in NZ (Health Promotion Agency [25])
Adult NZ population who downloaded a physical activity app, % (UI)	31 (21-41)	Beta	Estimated based on the proportion of survey respondents who had downloaded a physical activity app to track behavior (Krebs and Duncan [26])
Adult NZ population who used the physical activity app, % (UI)	16 (10-36)	Beta	Based on the proportion of people likely to “take action” after a UK-based mass-media campaign to promote app use (Brannan et al, [13])
Users who adhered to physical activity app (weighted annual average), % (UI)	15 (10-21)	Beta	Weighted average of estimates of “app only” adherence from Guertler et al [23]
Intervention increase in physical activity for those who adhered to the app (mins/week), n (SD)	285 (43)	Normal	Reported increase of 1404 steps per day from recent meta-analysis of randomized controlled trials (Gal et al [8]) was converted to MVPA^c^-MET^d^ mins/week, assuming a conversion factor of 34.5 steps equating to 1 MVPA-MET min [24]
Cost of a one-off national level mass media campaign (NZ $), n (SD %)	2,883,000 (20)	Gamma	As per a similar NZ study for promoting a weight loss app, by Cleghorn et al [19]; includes costs associated with identification of high-quality apps and mass media campaign across multiple media

^a^NZ: New Zealand.

^b^UI: uncertainty interval.

^c^MVPA: moderate-to-vigorous physical activity.

^d^MET: metabolic equivalent of task.

The total cost of the intervention was estimated at NZ $2,883,000 (using consumer price index and purchasing power parity adjustments, the currency exchange rate used for this paper was 2011 NZ $1=2018 US $0.73) from a previous NZ study of the costs associated with a modeled mass media campaign to promote a weight loss app [19]. The cost of the intervention captured the costs associated with identifying the highest quality apps, promotion of the highest quality apps for physical activity on relevant government websites, and a mass media campaign rolled out across multiple media. These costs were also similar to the estimated cost for a modeled mass media campaign to promote smoking cessation apps [18].

Increases in physical activity were applied to the proportion of the population who downloaded and used the app for at least 7 days. We assumed that the intervention effect would apply to adults 15-79 years of age. This was the population range covered in studies included in the review used to estimate physical activity increases in response to physical activity apps [8].

For those who used the app for at least 7 days, we estimated that physical activity would increase by an average of 285 moderate-to-vigorous physical activity–metabolic equivalent of task (MET) mins/week using a recent meta-analysis examining increases in physical activity associated with app use [8]. This is equivalent to 1.6 hours of additional brisk walking per week. We assumed that the intervention increase in physical activity would wane over the course of the year in which the intervention was implemented, with no effect beyond the first year of the intervention. This was in line with the source of our estimate of intervention increase in physical activity, where included studies were evaluated based on the short-term (<3 months) impacts.

BAU was assumed to include the existing levels of physical activity and existing levels of physical activity app use, with no additional promotion. The current promotion of physical activity apps in New Zealand was considered negligible, and therefore, the BAU physical activity distribution was assumed to reflect the continuation of a low or no physical activity app promotion environment.

### Multistate Life Table Model

**Figure 2:**
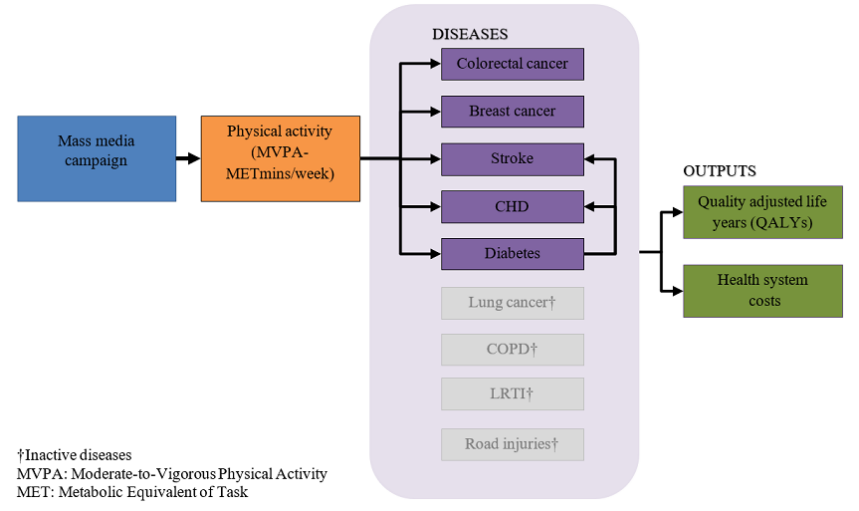
Conceptual diagram of model. CHD: coronary heart disease; COPD: chronic obstructive pulmonary disease; LRTI: lower respiratory tract infection.

The model consists of a main life table parameterized with age, sex, and ethnicity (Māori—the indigenous population of New Zealand—and non-Māori) specific all-cause mortality and morbidity rates. Alongside the main life table are 9 parallel physical activity and transport-related disease life tables where proportions of the population simultaneously reside: coronary heart disease (CHD), stroke, type 2 diabetes, colorectal cancer, breast cancer (females only), chronic obstructive pulmonary disease (COPD), lower respiratory tract infection (LRTI), lung cancer, and road transport injury. Modeled diseases include both physical activity and transport-related conditions, as the model was designed to examine both interventions. COPD, LRTI, lung cancer, and road transport injury were inactive (ie, “turned off”) in this study, as they are not associated with physical activity (see Figure 2 for the conceptual diagram, adapted from Mizdrak et al [20]). The proportions of the population in each disease life table at each annual time step are a function of past and current disease incidence, case fatality, and remission (for cancers only) rates.

The physical activity distribution of the New Zealand adult population was estimated by converting responses to the New Zealand Physical Activity Questionnaire Short Form in the New Zealand Health Survey to MET minutes per week of moderate and vigorous physical activity. A MET is the ratio of work metabolic rate to a standard resting metabolic rate, where 1 MET is equivalent to quiet sitting [27]. Brisk walking was assigned a MET value of 3.0, moderate activities a MET value of 4.5, and vigorous activities a MET value of 6.5 [20].

The modeled intervention induced changes in physical activity were combined with relative risks for the association between physical activity and disease outcomes (CHD, stroke, type 2 diabetes, breast cancer, colorectal cancer) to produce population impact fractions [28]. These were used in the model to modify incidence rates of diseases, which in turn results in changes in all-cause mortality and morbidity rates. The model includes time lags to account for the nonimmediate impact of changes in population risk distribution on disease incidence: changes in CHD, stroke, and type 2 diabetes are based on the average population impact fraction over the past 0-5 years, for cancers on average for the previous 10-30 years [20]. For modeling parsimony, we assumed that there would be no impact of the modeled intervention on health beyond that captured through the diseases previously mentioned, including no impact on obesity, injury, or mental health outcomes. These assumptions are consistent with the evidence base: there does not appear to be a consistent association with weight loss for apps that specifically target physical activity [29], and there is no evidence (to our knowledge) of the impact of physical activity apps on mental health outcomes or injury (as covered further in the Discussion).

In addition, the model captures changes in health system costs associated with changing disease prevalence and population longevity. Disease-specific costs were based on the timing of events (first year, subsequent year, and last 6 months of life) and were derived according to an established protocol [30]. Changes in the proportion of the population in each disease state result in proportional changes in health system costs, and the model captures unrelated health system costs (ie, increases in health system costs out into the future due to people living longer as a result of the modeled intervention).

Our results project the health gains and health system cost impacts for the remainder of the life course of the modeled population. Both health gains and health system costs were discounted at 3%, with key results using 0% and 6% discount rates presented as sensitivity analyses. We also ran the results applying an “equity adjustment” that set background mortality and morbidity rates for Māori to non-Māori values, a routinely used modeling technique that avoids undervaluation of health gains for disadvantaged populations [31]. Scenario analyses included a scenario where the age range for the intervention was restricted to those 40-79 years of age with total intervention costs remaining the same, and one in which we assumed that the intervention impact would be maintained for 5 years following the intervention. Finally, tornado plots show the contribution of assumptions around each step in the intervention pathway to model uncertainty of the results.

The model was built in Microsoft Excel (Microsoft Corporation) and run using a macro written in Visual Basic for Applications. Uncertainty around health gains and cost-effectiveness was estimated using a Monte Carlo analysis; the model was run 2000 times with parameters sampled independently from their respective probability distributions. Results are given as the 50th percentile of all model runs, with 2.5th and 97.5th percentiles representing the 95% uncertainty interval (UI) around modeled values. The probability of cost-effectiveness at different monetary thresholds was based on the proportion of model runs with an incremental cost-effectiveness ratio (ICER) below the threshold. Further model details are provided in a technical report [20].

## Results

**Figure 3:**
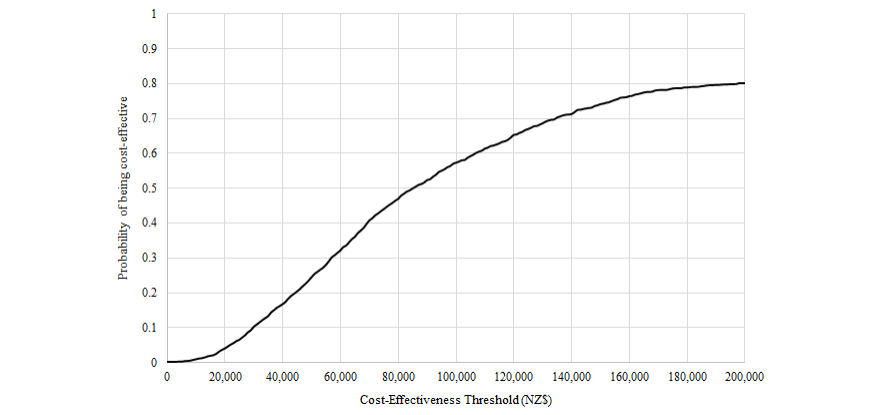
Probability of the modeled physical activity app promotion intervention being cost-effective for different cost-effectiveness thresholds (in cost per quality-adjusted life year gained).

The one-off mass media campaign promoting smartphone apps for physical activity resulted in an increase of 28 QALYs (95% UI 8-72) over the lifetime of the 2011 population, or 0.008 QALYs gained per 1000 people (see Table 2). The modeled improvements in health came at a net cost of NZ $2.2 million (US $1,625,000; 95% UI 1.02 million-3.5 million). The ICER was NZ $81,000 (US $59,000; 95% UI 17,000-345,000) per QALY gained. The intervention had a low probability (20%) of being cost-effective at a cost-effectiveness threshold of NZ $45,000 per QALY gained (see Figure 3).

Health gains per capita were higher in older age groups, and, assuming the intervention costs were spread evenly across the eligible population, the intervention was more likely to be cost-effective in older age groups compared to younger age groups (ie, more likely to be under the NZ $45,000 threshold). Health gains for Māori increased with the application of the “equity adjustment” (ie, non-Māori mortality and morbidity rates used for Māori; see Table 3).

**Table 2:** Health gains and cost-effectiveness of a mass media campaign to promote physical activity smartphone apps by age, sex, and ethnicity (lifetime gains, 3% discount rate).

Sex, ethnicity		Age group (years)	QALYs^a^/1000 population (UI^b^)	Cost per QALY gained: ICER^c^, 2011 NZ $ (UI)
All, all		All groups	0.008 (0.002-0.021)	81,000 (17,000-345,000)
**Male**				
	Non-Māori			
		<40	0.001 (0.000-0.003)	606,000 (190,000-2,368,000)
		40-60	0.008 (0.002-0.021)	86,000 (16,000-384,000)
		60-80	0.021 (0.006-0.055)	27,000 (cost-saving^d^ to 147,000)
	Māori			
		<40	0.002 (0.001-0.006)	354,000 (111,000-1,384,000)
		40-60	0.018 (0.005-0.047)	35,000 (2000-179,000)
		60-80	0.031 (0.009-0.083)	16,000 (cost-saving to 96,000)
**Female**				
	Non-Māori			
		<40	0.002 (0.000-0.005)	393,000 (120,000-1,499,000)
		40-60	0.006 (0.002-0.017)	119,000 (26,000-495,000)
		60-80	0.023 (0.007-0.061)	26,000 (cost-saving to 132,000)
	Māori			
		<40	0.003 (0.001-0.009)	196,000 (54,000-768,000)
		40-60	0.019 (0.005-0.049)	31,000 (0-158,000)
		60-80	0.035 (0.010-0.094)	15,000 (cost-saving to 87,000)

^a^QALY: quality-adjusted life year.

^b^UI: uncertainty interval.

^c^ICER: incremental cost-effectiveness ratio.

^d^Negative cost per QALY gained (ie, the intervention results in cost-savings to the health system).

**Table 3:** Results for Māori (Indigenous population) with equity adjustment applied (lifetime gains, 3% discount rate).

Sex, age (years)		QALYs^a^/1000 people (UI^b^)	Cost per QALY gained: ICER^c^, 2011 NZ $ (UI)
**Male**			
	<40	0.002 (0.001-0.006)	315,000 (92,000-1,191,000)
	40-60	0.022 (0.006-0.058)	30,000 (1000-142,000)
	60-80	0.046 (0.012-0.126)	11,000 (cost-saving^d^ to 69,000)
**Female**			
	<40	0.004 (0.001-0.010)	172,000 (43,000-669,000)
	40-60	0.024 (0.006-0.062)	26,000 (cost-saving to 130,000)
	60-80	0.052 (0.014-0.137)	10,000 (cost-saving to 64,000)

^a^QALY: quality-adjusted life year.

^b^UI: uncertainty interval.

^c^ICER: incremental cost-effectiveness ratio.

^d^Negative cost per QALY gained (ie, the intervention results in cost-savings to the health system).

We explored the impact of selected changes to model specification on the results (see Table 4). Given that the intervention was least cost-effective in the youngest age group, we ran a scenario analysis to determine the extent that the overall cost-effectiveness might be improved by narrowing the population targeted by the intervention to those 40-80 years of age. This slightly increased the average cost-effectiveness of the intervention. Assuming the impact of the intervention held for 5 years rather than 1 year, the health gains would be over four times larger than in the main analysis and would result in much lower health system costs, resulting in a highly cost-effective ICER of NZ $2000 per QALY gained. Changing the discount rate had the expected impact on the overall results, with a zero-discount rate resulting in higher health gains.

**Figure 4:**
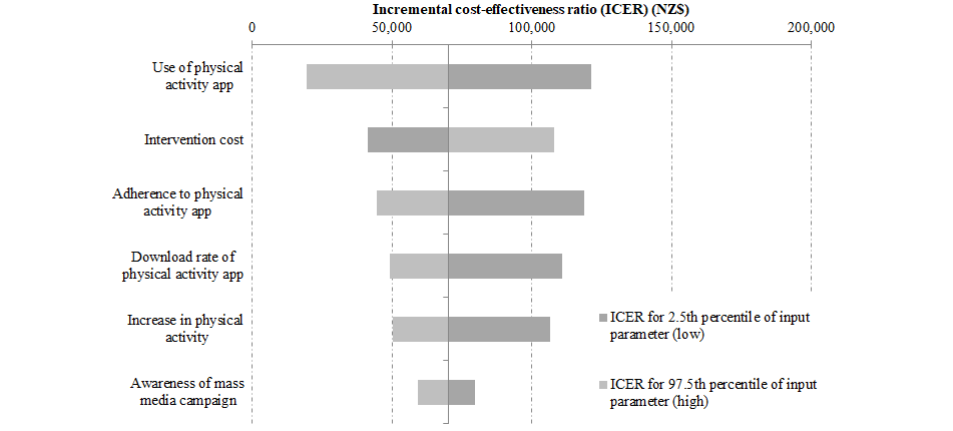
Tornado plot showing the contribution of parameter uncertainty to overall uncertainty in the incremental cost-effectiveness ratio for the whole adult population.

Finally, we examined the contribution of different intervention parameters to uncertainty in the modeled results. Uncertainty in health gains was driven by uncertainty in the app use parameter, and uncertainty in health system cost impacts was driven by uncertainty in the intervention cost parameter (see Figures S1 and S2 in Multimedia Appendix 1). The picture for the ICER was less clear; uncertainty in app use was the greatest contributor to uncertainty in the ICER, but this was closely followed by uncertainty around other intervention parameters (see Figure 4).

**Table 4:** Sensitivity and scenario analyses for a one-off national-level mass media campaign to promote smartphone apps for physical activity (expected value analysis, lifetime perspective, 3% discount rate, unless otherwise stated).

Sensitivity/scenario analyses^a^	Health gain (QALYs^b^)	Net health system costs (NZ $)	Cost per QALY gained: ICER^b^ (NZ $)
Base case analysis	33	2,315,000	81,000
Target age range set to 40-80 years of age (otherwise base case)	30	2,387,000	80,000
5-year maintenance of additional physical activity levels followed by a return to preintervention levels (otherwise base case)	126	241,146	2000
0% discount rate	57	2,153,000	38,000
6% discount rate	22	2,332,000	108,000

^a^Expected values given for all scenarios.

^b^QALY: quality-adjusted life year.

^c^ICER: incremental cost-effectiveness ratio.

## Discussion

### Principal Findings

We modeled the likely impact of a one-off national-level mass media campaign to promote uptake of smartphone apps for physical activity using published estimates of uptake, adherence, and effectiveness [8,13,23,25,26]. Modeled through changes in disease incidence the intervention has a 20% chance of being cost-effective for the whole target population at a commonly applied threshold of GDP per capita of the country (ie, NZ $45,000 per QALY gained for New Zealand) [32]. There was also wide uncertainty around the health system cost impacts and cost-effectiveness of the intervention.

### Comparison With Prior Work

This is the first study of the cost-effectiveness of mass media promotion of smartphone apps for physical activity, at least that we are aware of. This study contributes to calls to build the evidence base on the cost-effectiveness of physical activity interventions [33]. This work also has a high level of comparability with previous research on other health-related app promotion in the NZ setting. We found that a mass media campaign to promote physical activity apps appears to be less effective in achieving health gain and less cost-effective than mass media campaigns to promote smoking cessation (modeled impact: 6760 QALYs, NZ $115 million savings to the health system [18]) but was similar to a campaign to promote the use of weight loss apps (modeled impact: 29 QALYs, ICER of NZ $79,700 [19]). This suggests that mass media campaigns to promote apps may have different impacts depending on the behavior targeted by apps.

Our results also indicate lower effectiveness and poorer cost-effectiveness on a per capita basis than previous research that modeled the effectiveness of a mass media campaign and other strategies to promote physical activity in Australia [34]. This is likely due to our study applying more conservative estimates for the impact of a mass media campaign intervention than earlier work and differences in underlying physical activity patterns and epidemiology across different countries.

### Strengths and Limitations

This study has the strength of using an established multistate life table model based on rich disease-specific epidemiological and costing data. Multistate life table modeling captures health impacts across multiple diseases over time. The widespread use of this modeling methodology across Australasia means we are able to compare our results to those of other health interventions (eg, [18,19,34]). Limitations of multistate life table modeling include the assumption of disease independence and our use of a health-system perspective for costs and benefits. Regarding the former, we do account for the relationship between type 2 diabetes and CHD and stroke, given that type 2 diabetes is a risk factor for these conditions. The health system perspective of this study means that potential costs and benefits outside the health system (eg, the cost of the original development of physical activity apps) were not captured. However, our methods could be adapted to include additional costs and benefits for different audiences. For example, we are currently exploring the inclusion of productivity impacts, such as income loss from disease diagnosis, into our models.

Effect sizes were based on a review of relevant sources that were then assessed on both methodological rigor and appropriateness for modeling (see Multimedia Appendix 1 for further details). We also were able to model parameter uncertainty around all the key parameters and a range of sensitivity and scenario analyses.

We presented heterogeneity in the health impacts of the modeling intervention. As we assumed no heterogeneity in intervention impact, our estimates reflected differential health gains owing to underlying differences in physical activity levels and epidemiology, and not differential response to the intervention. There was insufficient information in the sources of parameter estimates to suggest differences in intervention impacts by age, sex, or ethnicity. Previous research has shown high levels of engagement with physical activity apps across subpopulations, including older adults [35] and different ethnic groups [36]. However, if certain population groups are more or less likely to respond to the intervention, then this would influence the overall effectiveness and cost-effectiveness. Future evaluations of physical activity app interventions should explicitly consider differential impacts in intervention uptake and efficacy, especially given that our results showed differences in the health gain likely to be achieved.

We assumed that any impact of the intervention on physical activity levels would be restricted to the year in which the intervention was implemented, consistent with existing evidence. The majority of physical activity apps have only been evaluated for short-term (<3 months) impact [7,8]. Our estimate of average adherence in the year that the app was implemented is consistent with the small number of studies that have evaluated physical activity impacts beyond 3 months [23,37]. There was no evidence to support modeling an intervention impact that extended beyond 1 year, and this highlights the need for longer-term evaluations of physical activity app interventions, especially as our scenario analysis demonstrates that considerably larger health gains could be achieved if intervention impacts were maintained over time. Evidence of long-term impacts on physical activity have been observed with interventions to improvement in walking and cycling infrastructure [38,39], suggesting that structural interventions that change environments may be more effective than interventions targeting individual-level behavior change in an unsupportive environment.

The model captures health impacts through the conditions that are strongly associated with MVPA including cardiovascular diseases, type 2 diabetes, and selected cancers. We did not capture potential additional health gains or losses from obesity, mental health, or injury. Although apps have been shown to be effective in promoting weight loss [40], effect sizes predominantly capture apps designed to influence both physical activity and dietary behaviors. For apps that specifically target physical activity, there does not appear to be a consistent association with weight loss [29]. In addition, the impact of physical activity apps on mental health outcomes has not been quantified (to our knowledge). Although regular physical activity is associated with improved mental health [41], recent evidence suggests that a high percentage of fitness apps contain features linked to negative body image and maladaptive exercise behavior [42]. Further research is needed to understand whether health gains associated with increased physical activity through app use are complemented or counteracted by other health-related outcomes, including weight loss, injury, and mental health.

### Policy Implications

The wide UIs around our modeled results demonstrate the need for better evaluations of app-based and other physical activity interventions. In particular, we need to better understand what interventions are most likely to result in long-term maintenance of physical activity increases, as these are the interventions that will result in the largest health gains. Modeling studies such as this one are a valuable approach to quantify the health gains that may be possible with different intervention options prior to implementing specific interventions.

Although physical activity apps offer the potential to increase physical activity at the individual level, our results suggest that promoting physical activity apps through mass media is currently unlikely to be an effective or cost-effective public health intervention at the population level, at least with existing app designs and mass media campaign methods. Cost-effectiveness of a mass media campaign to promote smartphone apps for physical activity could be improved by delivering more targeted campaigns using social media; this may deliver health gains at lower cost than the intervention modeled here. Worldwide, there is recognition that targeted, individual-focused interventions need to be combined strategically with policy actions that support physical activity [41]. Our results suggest that a mass media campaign to promote smartphone apps for physical activity is not cost-effective as a stand-alone intervention. Other strategies to promote physical activity that result in long-term behavior change are likely to be more effective (eg, investment in walking and cycling infrastructure [38]). Our results are likely to be generalizable to similar contexts—high-income countries with similar epidemiology, physical activity levels, app uptake, and other population characteristics.

### Conclusion

A one-off national-level mass media campaign to promote the use of smartphone apps for physical activity is unlikely to generate much health gain. Based on current and often weak evidence, it also appears unlikely to be cost-effective at the population level. Investments in physical activity that are associated with long-term maintenance of behavior are likely to be of greater benefit.

## Appendix

Multimedia Appendix 1 Supplementary materials.

## Abbreviations

BAU: business-as-usual
CHD: coronary heart disease
COPD: chronic obstructive pulmonary disease
ICER: incremental cost-effectiveness ratio
LRTI: lower respiratory tract infection
MET: metabolic equivalent of task
MVPA: moderate-to-vigorous physical activity
QALY: quality-adjusted life years
UI: uncertainty interval
