# Supplementary material for: Health Gain, Cost Impacts, and Cost-Effectiveness of a Mass Media Campaign to Promote Smartphone Apps for Physical Activity: Modeling Study
Source: JMIR Mhealth Uhealth. 2020 Jun 11;8(6):e18014. doi: 10.2196/18014 (PMC7317635; doi:10.2196/18014)

# Supplementary Materials

Figure S1: Tornado plot showing the contribution of parameter uncertainty to overall uncertainty in the quality-adjusted life years gained for the whole adult population


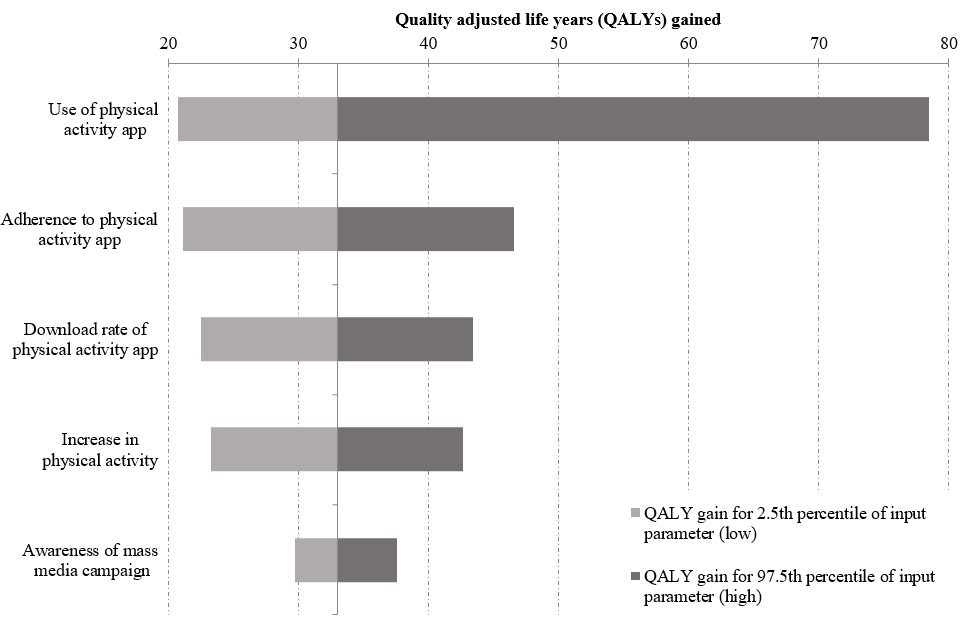


Figure S2: Tornado plot showing the contribution of parameter uncertainty to overall uncertainty in health system costs for the whole adult population


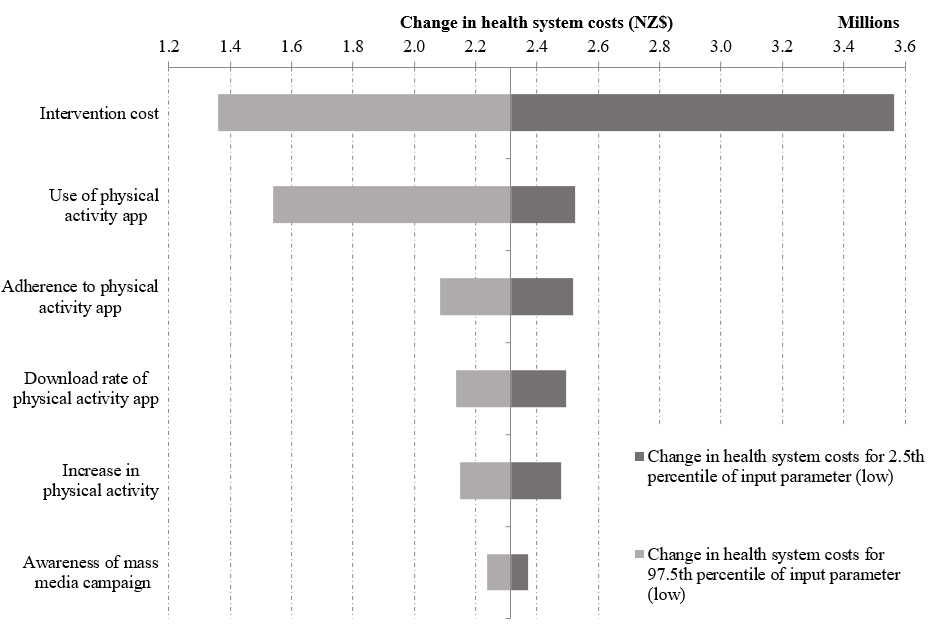

Supplement: Multimedia Appendix 1 [file mhealth_v8i6e18014_app1.doc]
